# Immobilization of Potentially Toxic Elements in Contaminated Soils Using Thermally Treated Natural Zeolite

**DOI:** 10.3390/ma14143777

**Published:** 2021-07-06

**Authors:** Oana Cadar, Zamfira Dinca, Marin Senila, Anamaria Iulia Torok, Florin Todor, Erika Andrea Levei

**Affiliations:** 1 INCDO-INOE 2000, Research Institute for Analytical Instrumentation, 67 Donath Street, 400296 Cluj-Napoca, Romania; oana.cadar@icia.ro (O.C.); zamfira.dinca@icia.ro (Z.D.); iulia.torok@icia.ro (M.S.); marin.senila@icia.ro (A.I.T.); 2 SC MINESA ICPM SA, 15-17 Tudor Vladimirescu Street, 400225 Cluj-Napoca, Romania; minesa.laborator@gmail.com

**Keywords:** natural zeolite, thermal treatment, soil, immobilization, toxic elements

## Abstract

Rehabilitation of contaminated soils is a complex and time-consuming procedure. One of the most cost-effective and easy-to-use soil remediation approaches is the use of amendments that stabilize the potential toxic elements (PTE) in soil by reducing their mobility and bioavailability. The stabilization of Cu, Pb, Zn, Cd, Co, Cr, Ni in a contaminated soil using 5% and 10% amendment with thermally treated natural zeolite was investigated using a sequential extraction procedure, contamination and environmental risk factors. The results showed that after amendment, the PTE concentration decreased in the exchangeable and reducible fractions and increased in the oxidizable and residual fractions. The highest immobilization effect, consisting in the decrease of exchangeable fractions with 69% was obtained in case of 10% zeolite amendment and 90 days of equilibration time for Pb; also, more than half of the mobile fraction was immobilized in case of Zn, Cu, and Co and about one third in case of Ni, Cr, and Cd. Generally, the immobilization effect of the 5% and 10% amendment is comparable, but a higher equilibration time enhanced the immobilization effect, especially in the case of Cd, Co, Cu, Pb, and Zn.

## 1. Introduction

Soil pollution is a type of land degradation that takes place when a range of natural or anthropogenic constituents exceed the concentrations normally found in natural soils, affecting its quality and ecological services [1]. Generally, the main anthropogenic sources of soil pollution are represented by industry, agriculture, and household activities [2].

The potential toxic elements (PTE) are a group of metals and metalloids that can cause long-term health risks to humans, animals, plants, and ecosystems [3]. Some of the PTEs naturally exist in soils at low concentrations, and they are essential micronutrients for plants, but may cause toxic effects in large quantities. Moreover, since most PTEs are non-degradable, they persist in the environment for a long time [4]. In different environmental conditions, PTE may be leached to the surrounding river systems or groundwater, affecting their quality [5].

In the last few years, the remediation of PTE-contaminated soil received growing interest, due to the increasing land degradation and decreasing productive lands [6,7]. Numerous in situ and ex situ soil remediation techniques have been used to diminish the risks associated with PTE contamination to maximize the surfaces suitable for agriculture, thus guaranteeing food security [8]. Among in situ methods, the application of soil amendments to immobilize PTEs has been broadly used, due to its fast and easy application and commercial viability. Immobilization is a process which puts the PTE into a chemical form which will behave as inert to biological systems and highly insoluble under normally existing conditions [2,9]. The use of suitable immobilizing agents can lead to cost-effective and efficient soil remediation [8,10]. In this regard, the remediation using amendment with reactive minerals (carbonates, phosphate rocks, clay minerals, and zeolites) reduces the bioavailability of PTE, often without significantly altering the natural functions of soil [11]. Some of these amendments modify the soil pH, leading to precipitation of insoluble phases containing PTE, while others immobilize the PTE by sorption or encapsulation in their crystalline structure [2].

Natural zeolites are crystalline, hydrated aluminosilicates consisting of three-dimensional frameworks of [SiO_4_] and [AlO_4_] tetrahedra connected to each other through oxygen bridges [12]. Recently, natural zeolites and their modified forms have received considerable attention due to their remarkable physical and chemical properties (hydration-dehydration behavior, adsorption of molecules, ion exchange ability without any structural modification, etc.), accessible source, and low cost. Zeolites are presently widely used in industry, agriculture, and pollution control [13,14]. Natural zeolites have been used for PTE immobilization plain or in association with other minerals (clay minerals) or other decontamination processes, such as phytoremediation. Besides, natural zeolites slightly adjust pH and do not introduce additional pollution in the environment [15].

Natural zeolite deposits exist in many countries, but the world reserves of natural zeolites have not yet been estimated. The price for natural zeolite depends on the purity, mineralogy, type of processing, and type of application, and ranges between 50–300 $/metric ton, while synthetic zeolites are significantly more expensive [16,17]. More than 70 natural zeolite types exist worldwide and more than 260 zeolites have been synthesized. In Romania, 15 natural zeolite types were identified; however, the exploitation and valorization of the zeolite deposits is low [18]. Previous laboratory-scale investigations and field tests indicated that natural zeolites and their modified forms decrease the concentration of PTE and other hazardous substances in soils, limit the ground erosion and maintain the organic-matter content. Contin et al. reported that PTE mobility was reduced by the addition of 2.5% *w*/*w* natural zeolite, while the addition of 10% *w*/*w* natural zeolite immobilized the PTE [7]. Due to the wide variety of natural zeolites, it is difficult to specify a universal zeolite application rate that ensures an efficient PTEs immobilization. The climatic and environmental conditions together with the soil and zeolite type plays an important role in the PTE immobilization efficiency [2,15]. The long-term effects of natural zeolites on soil pH and essential metals’ availability, the mechanism of Na release from zeolites, or the PTE binding to zeolite are not fully understood. Moreover, the reported experiments on metals’ immobilization by natural zeolite addition displayed variable results. A possible explanation could be the wide variation of the zeolites’ cation exchange capacity (CEC) determined by the different cage structure, structural defects, adsorbed ions, and presence of gangue minerals [7]. Due to the insufficient data, it is difficult to specify a universal natural zeolite application rate. Therefore, more in-depth experiments on natural zeolite application rates need to be conducted in order to gain insights on short- and long-term behavior and to identify the appropriate doses for pre-defined aims.

This study aims to investigate the use of Macicas natural zeolite for soil decontamination. The immobilization of PTEs (Cd, Cr, Co, Cu, Ni, Pb, and Zn) by a thermally treated natural zeolite in industrially contaminated soils was tested in pot experiments using different amendment rates (5 and 10 wt.%) and equilibration periods (30 and 90 days). The PTE mobility before and after the amendment was assessed by the BCR sequential extraction procedure.

## 2. Materials and Methods

### 2.1. Zeolite Collection and Preparation

Raw zeolite (RZ) was collected as rocks from Macicas area, Cluj County, Romania. The RZ was crushed and sieved to obtain a particle size <1 mm, dried in an oven at 105 °C and then thermally treated at 200 °C for 2 h in air. For the immobilization experiments, the thermally treated zeolite (TZ) was used.

### 2.2. Soil Collection and Preparation

Lower Iara Valley Basin is known for its deposit of magnetite mineralization associated with garnet and pyroxene skarns, limestone, and metalliferous deposits rich in precious metals [19]. Due to legacy mining, there are several tailing deposits containing silicates in this area (garnets, pyroxenes, amphiboles, etc.), carbonates (dolomite and subordinately calcite) and metallic minerals (sulfides and oxides) [19]. Weathering processes favors the oxidation–hydration of minerals from tailings leading to the pollution of the environment. A composite soil sample containing the upper (0–10 cm) soil layer was randomly collected from a surface of 100 m × 100 m from a former mining area in Iara, Cluj County, Romania, using a stainless-steel shovel. After the removal of vegetation residues and stones, the sample was thoroughly homogenized. For chemical characterization, an aliquot of the soil sample was air-dried, ground, and sieved through a 2 mm sieve to remove gravels.

### 2.3. Soil Amendment with Zeolite

The soil was amended with 5% and 10% *w*/*w* (dry weight basis) thermally treated zeolite (TZ) and thoroughly mixed in 2 L pots. The obtained mixtures containing 5% *w*/*w* TZ (TZS5) and 10% *w*/*w* TZ (TZS10) were moistened until saturation point with distilled water and left for equilibration for 30 (TZS5-30, TZS10-30) and 90 days (TZS5-90, TZS10-90). Additionally, the pots were watered periodically with distilled water to maintain soil moisture. A control (C) pot containing soil without amendment was treated similarly with the test pots. Each experiment was performed in triplicate. At day 0, day 30, and day 90, samples were collected from each pot, dried at 60 °C for 48 h in a universal oven (UFE 400, Memmert, Schwabach, Germany) and subjected to analysis.

### 2.4. PTEs Immobilization in Zeolite Treated Soil

As by amendment with zeolite, the PTEs are not removed from soil, the immobilization efficiency was evaluated in terms of PTEs leachability using the modified BCR procedure. This procedure is a useful tool to predict short- and long-term mobility of trace elements, mimicking complex environmental scenarios [20]. The modified BCR sequential extraction procedure described by Pueyo et al. [20] was applied to a 1 g sample from both test and control experiments in order to investigate the change of PTE leachability, under the influence of amendment and equilibration time. Briefly, the PTEs were separated into four operationally defined fractions: F1-Exchangeable and weak acid soluble fraction, that contains the PTEs extracted in 0.11 M acetic acid, F2-Reducible fraction, containing the PTEs associated mainly with Fe and Mn oxides, extracted in hydroxylamine, F3-Oxidizable fraction containing PTEs bonded to organic matter, extractable with H_2_O_2_ and NH_4_OAc and F4-Residual fraction containing PTEs soluble in aqua regia. The detailed description of the fractionation procedure is presented by Frentiu et al. [21]. The accuracy of the sequential extraction method was assessed using the % recovery of the total metal concentration as the sum of fractions F1–F4. The recovery rates for studied elements ranged between 89.6% and 108%.

### 2.5. Physico-Chemical Analysis

All reagents were of analytical grade (Merck, Darmstadt, Germany) and were used as received without further purification. Ultra-pure water from a Purelab Flex 3 system (Buckinghamshire, UK) was used for all dilutions and for the preparation of standard solutions.

The pH was measured in a 1:5 solid: water (*w*:*v*) suspension using a Seven Excellence multiparameter (Mettler Toledo, Schwerzenbach, Switzerland) [6]. The cation exchange capacity (CEC) of soil was determined by the NH_4_Cl–NH_4_COOH method described by Ciesielski [22] and of zeolites by the modified ammonium acetate saturation (AMAS) method reported by Kitsopoulos [23]. The X-ray diffraction (XRD) patterns were recorded at room temperature using a D8 Advance (Bruker, Karlsruhe, Germany) diffractometer with CuKα radiation (λ = 1.54060 Å), operating at 40 kV and 40 mA. The SiO_2_ and loss of ignition (LOI) were determined using the gravimetric method [18]. The total carbon (C_T_) and nitrogen (N_T_) content were performed using a Flash 2000 CHNS/O analyzer (Thermo Fisher Scientific, Waltham, MA, USA) by combustion of a 2–3 mg sample; the instrument calibration was performed with atropine (70.56% C and 4.84% N, Thermo Fisher Scientific, Waltham, MA, USA) [24]. Humic (HA) acids were determined following the procedure proposed by Ciavatta [25].

For the metals analysis, the zeolite samples were digested with a mixture of HNO_3_ 65%: HCl 37%: HF 40% (3:9:2, *v*:*v*) in a closed-vessel Speedwave Xpert microwave system (Berghof, Eningen, Germany) using the method described previously [26,27]. The soil sample was digested in aqua regia (HCl 37%: HNO_3_ 65%, 3:1, *v*:*v*), filtered and diluted to 100 mL with ultra-pure water [28]. The metal contents were determined using Optima 5300 DV (Perkin-Elmer, Woodbridge, ON, Canada) inductively coupled plasma-optical emission spectrometer (ICP-OES). The conversion of the metal content to the corresponding oxide content was made using atomic and molecular masses.

The Cd, Cr, Co, Ni, and Pb concentrations in the F1–F4 fractions were determined using the inductively coupled plasma mass spectrometer ICP-MS ELAN DRC II (Perkin-Elmer, Toronto, ON, Canada), while Cu and Zn concentrations by Optima 5300 DV (Perkin-Elmer, Woodbridge, ON, Canada) ICP-OES.

The calibration curves were prepared using the 1000 mg/L multi-element (Al, Fe, Ca, Mg, K, Na, Mn, Cd, Cr, Co, Cu, Ni, Pb, and Zn) and mono-element (Ti) standard solutions (Merck, Darmstadt, Germany) for ICP-OES and the 10 mg/L multi-element standard solution III (Perkin Elmer, Norwalk, CT, USA) for ICP-MS.

A potash feldspar (BCS-CRM 376/1, Bureau of Analyzed Samples, Middlesbrough, UK) with a similar matrix to zeolite samples, Loam Soil (ERM-CC141, Institute for Reference Materials and Measurements, Geel, Belgium) and BCR 701 (lake sediment certified for BCR three-step sequential extraction, IRMM, Geel, Belgium) certified reference materials were analyzed for quality control purposes. Acceptable accuracy (80–120%) and precision (≤20%) of metals determination was obtained.

### 2.6. Calculation of Environmental and Risk Factors

To study the PTE mobility reduction after zeolite amendment, the individual contamination factor (*ICF*) and the global contamination factor (*GCF*) were calculated for both unamended and zeolite-amended soils. The *ICF* indicates the risk posed by PTEs, as high values of *ICF* indicate low retention of PTEs in soil and was calculated for each PTE by dividing the sum of mobile and potentially mobilizable fractions by the residual fraction (Equation (1)). *ICF* values ≤ 1 indicate low contamination, 1–3 moderate contamination, 3–6 considerable contamination, and >6 high contamination [29,30]. The *GCF* indicates the multi-element contamination and was calculated according to Equation (2) [31]. Soils are considered to have low global contamination if *GCF* < 6, moderate global contamination if the *GCF* ranges between 6–12, considerable global contamination at *GCF* values between 12–24, and highly contaminated at *GCF* > 24 [31].

(1) $$ICF=\frac{F1+F2+F3}{F4}$$

(2) $$GCF=\overset{7}{\underset{i=1}{\sum }}ICFi$$

where *ICF* is the individual contamination factor, *F*1 is the individual PTE concentration in the exchangeable and weak acid soluble fraction, *F*2 is the individual PTE concentration in the reducible fraction, *F*3 is the individual PTE concentration in the oxidizable fraction, *F*4 is the individual PTE concentration in the residual fraction, and *GCF* is the global contamination factor.

The risk assessment code (*RAC*) is calculated as the percentage of the total PTE concentration that was found in the first fraction of the BCR method (Equation (3)) and indicates the environmental risk posed by PTEs [29,32].

(3) $$RAC=\frac{F1}{F1+F2+F3+F4}\times 100$$

where *RAC* is the risk assessment code, *F*1 is the individual PTE concentration in the exchangeable and weak acid soluble fraction, *F*2 is the individual PTE concentration in the reducible fraction, *F*3 is the individual PTE concentration in the oxidizable fraction, and F4 is the individual PTE concentration in the residual fraction.

The higher the *RAC* value, the greater is the risk associated to these elements, as it indicates the most bioavailable fraction of the PTEs. *RAC* values < 1% indicate no risk, 1–10% reflect low risk, 11–30% medium risk, and 31–50% high risk. Above 50%, the soils pose a very high risk and is considered dangerous, with mobile PTEs that easily enter in the food chain [29,32].

The environmental risk factor (*ERF*) classifies the risk posed by PTEs based on the first two fractions of the BCR extraction procedure, considered to have the highest mobility (Equation (4)). *ERF* values < 0.4 indicate low risk, 0.4–1 medium risk, and higher than 1 indicate high risk [30,32].

(4) $$ERF=\frac{F1+F2}{F3+F4}$$

where *ERF* is the environmental risk factor, *F*1 is the individual PTE concentration in the exchangeable and weak acid soluble fraction, *F*2 is the individual PTE concentration in the reducible fraction, *F*3 is the individual PTE concentration in the oxidizable fraction, and F4 is the individual PTE concentration in the residual fraction.

## 3. Results and Discussion

### 3.1. Properties of Raw and Thermally Treated Zeolite

The physico-chemical properties of the raw (RZ) and thermally treated (TZ) zeolites are presented in Table 1. The Macicas natural zeolite occurs as zeolitic-rich tuffs with zeolitic composition accompanied by impurities. According to XRD analysis, both the RZ and TZ consisted of clinoptilolite and plagioclase accompanied by minor quantities of quartz, modernite, and muscovite. Clinoptilolite is considered an excellent adsorption and ion exchanger material. The selectivity of cation exchange in case of zeolites with clinoptilolite content was reported to decrease in the following order: Pb^2+^ > Cd^2+^ > Cs^+^ > Cu^2+^ > Co^2+^ > Cr^3+^ > Zn^2+^ > Ni^2+^ > Hg^2+^ [33,34].

In accordance with the chemical composition, the RZ and TZ samples contain mainly Ca as exchangeable cation and smaller quantities of Na, K, and Mg. The theoretical CEC values calculated based on the chemical analysis are 244 meq/100 g (RZ) and 248 meq/100 g (TZ), that far exceeded the effective CEC value determined by the AMAS method, meaning 148 meq/100 g (RZ) and 150 meq/100 g (TZ). These results suggested that almost 60% of exchangeable sites are active [36]. The zeolite has alkaline pH and the PTEs concentration is very low, ranging from 0.16 mg/kg (Cd) to 15.9 mg/kg (Zn); thus, the amendment does not increase the soil contamination. The high content of clinoptilolite, high CEC, and low PTE concentration makes the RZ and TZ suitable to be used as amendment for contaminated soils, in order to reduce the share of mobile PTE fraction.

The XRD data of TZ indicated that the thermal treatment at 200 °C does not produce observable structural changes. The zeolite thermal stability at low temperatures can be explained by the reversible dehydration that takes place with little or no modification of the crystal structure [37]. Natural zeolites can be dehydrated without destruction of the crystal structure and rehydration through water adsorption. Generally, the water content in cages and channels of the zeolite framework is about 10–25% [38]. Thermal treatments allow the removal of impurities and modify the zeolite’s characteristics by controlling their structural and morphological properties [38]. Depending on the zeolite type and thermal treatment temperature, it increases the pore volume and surface area by removing the water and organic molecules from pore channels. Thus, the thermal treatment is a simple and green method that enhances adsorption capacity and specific surface area. No important changes in the physico-chemical properties of TZ were observed.

### 3.2. Soil Characteristics

**Figure 1:**
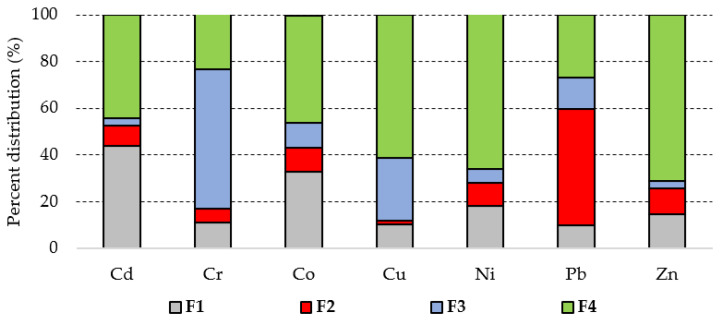
Potentially toxic elements (PTE) distribution between exchangeable and weak acid soluble fraction (F1), reducible fraction (F2), oxidizable fraction (F3), and residual fraction (F4) in soil.

The XRD data indicated that main mineralogical phases in the soil sample are quartz, calcite, and dolomite (most abundant), muscovite, microcline, kaolinite, gypsum, and gismondine. The metal concentrations in soil decrease in the order Zn >> Pb > Cu >> Cd > Co > Ni > Cr. The soil pH was slightly alkaline, the Cr, Co, and Ni concentrations were low, while those of Cd, Cu, Pb, and Zn exceeded the Romanian intervention thresholds for sensitive soil use (Table 1). As seen in Figure 1, Cd and Co are found predominantly in exchangeable and residual forms, Pb is bounded mainly to carbonates, Cr is mainly bounded to Fe and Mn oxides, while Cu, Ni, and Zn are found mainly in residual fractions. Generally, the PTEs in the first two fractions are considered to be the most easily mobilizable and bioavailable with a high possibility to enter in the food chain [11]. More than half of Cd (53%) and Pb (60%), almost half of Co (43%), and around a quarter of Ni (28%) and Zn (26%) concentrations are in fractions F1 and F2 and are easily mobilizable. The mobilizable fractions of Cr and Cu are low (<20%). The high total Cd and Pb concentrations, together with their high percentage of easily mobilizable fractions and their high toxicity may pose important environmental threats.

The recovery calculated as the % of total PTE found in the sum of fractions were 95% for Cd, 86% for Cr, 102% for Co, 94% for Cu, 89% for Ni, 97% for Pb and 103% for Zn. The recoveries ranging between 86% and 103% indicate an appropriate accuracy of the sequential extraction procedure.

### 3.3. PTE Immobilization

The pH in the zeolite-amended soils slightly increased after the stabilization period, however the pH increase was not influenced by the amendment ratio (Table 2).

The content of C_T_, N_T_, and humus did not change significantly during the experiment. The CEC was similar after amendment with 5% zeolite and slightly increased after amendment with 10% zeolite. During the stabilization period, the CEC further increased in both experiments, but did not change in the control sample. The increase of soil CEC with the increasing amount of added zeolite and the increase of equilibration time, from 61.4 meq/100 g (C) to 76.7 meq/100 g (TZS5-90) and 79.0 meq/100 g (TZS10-90) suggests that the addition of zeolite favors the cation exchange. However, the rise in zeolite amendment dose did not determine a significant CEC increase.

The distribution of PTEs between fractions with different mobility is influenced by the PTE nature and concentration, as well as the soil properties, such as pH and CEC. Following amendment, the soil pH and CEC are modified and thus, the PTE mobility in soil is expected to decrease [39]. As expected, the amendment with zeolites neither changes the total metal concentration, nor the sum of the four fractions. A possible explanation could be that the added amendments were low (5% and 10%), the dilution effect determined by the amendment being in the method determination error range.

The PTE concentrations during the immobilization experiments are presented in Table 3. The PTEs concentration in the fractions did not change in any of the two amendments, nor during the equilibration time. The percent distribution of PTEs in the control experiments are similar with that of the initial soil, Cd and Co being found mainly in F1 and F4 fractions, Cr in F3, Cu, Ni, and Zn in F4, while Pb in F2. Changes in the PTE concentration in the fractions were observed in case of both 5% and 10% amendments.

Generally, the PTE concentration increased in fractions F3 and F4 and decreased in F1 and F2. In case of the 5% amendment, F1 decreased with 19–37% and 31–57% after 30 and 90 days of equilibration, respectively. The highest decrease of F1 was observed for Ni (37%) after 30 days and for Pb (57%) after 90 days, while the lowest decrease was observed for Cd (19%) after 30 days and for Co (32%) after 90 days of equilibration. PTEs in fraction F1 can displace cations as K, Ca, Mg that are weakly associated in the zeolite structure. In F2, Co and Cu concentrations decreased with more than 20%, the other PTEs concentration remaining more or less constant, both after 30 and 90 days of equilibration. Important increases of Cd, Co, Ni, and Zn concentrations were observed in F3, but not in F4, while Pb and Cr remained more or less constant in F3, but increased in F4.

A similar behavior was observed in case of the 10% amendment, but the decrease of F1 was higher (18–46% after 30 days and 22–69% and 90 days of equilibration). The highest decrease of F1 and thus the best immobilization was observed for Cu (46%) after 30 days and for Pb (69%) after 90 days, while the lowest decrease was observed for Ni, that decreased with 18% after 30 days and with 22% after 90 days of equilibration. The Zn and Cu concentrations in F1 also decreased to half after 90 days of equilibration. In F2, only the Cu concentration decreased with more than 20%, while the rest of the PTEs remained more or less constant after 30 days; after 90 days of equilibration, the Co, Cu, Pb, and Zn concentrations decreased with more than 20%. The high immobilization rate of Pb (69%) could be explained by the higher selectivity of cation exchange in case of zeolites with clinoptilolite content for Pb^2+^ than for other PTEs [34,36]. A similar Pb solubility decrease was obtained by Moirou et al. [36] in case of the stabilization of contaminated soils from a mining area in Montevecchio, Sardinia, using clinoptilolite rich tuff from Pentalofos, Evros. Important increases of the Cd, Co, Cu, Pb, and Zn concentrations in F3 were observed after 30 days, while after 90 days of equilibration, the Cd and Zn concentrations doubled in F3 similarly, to the 5% amendment, while Pb and Cr almost doubled in F4. These results indicate that the zeolites may exchange cations with PTEs and thus change their mobility.

**Figure 2:**
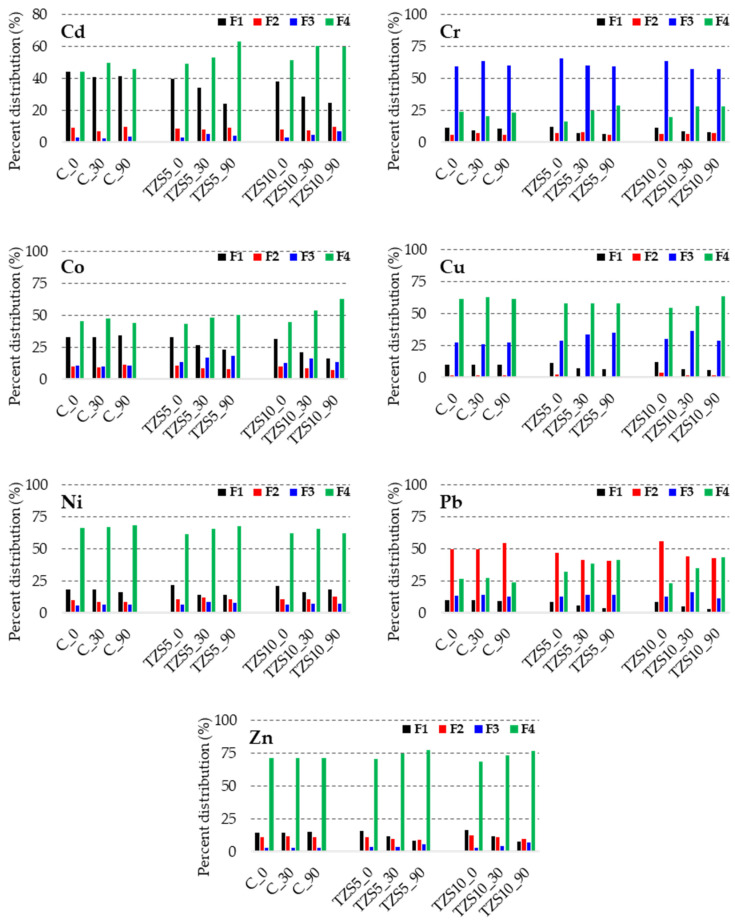
Percentage distribution of potentially toxic elements (PTE) concentrations in exchangeable and weak acid soluble fraction (F1), reducible fraction (F2), oxidizable fraction (F3) and residual fraction (F4) in the control soil (C) and soil amended with 5% (TZS5) and 10% (TZS10) zeolite at 0, 30, and 90 days after amendment.

The PTE distribution pattern (Figure 2) confirms the absence of changes in the control experiment for every studied PTE and the partial immobilization of PTEs after amendment with 5% and 10% zeolite. The mobility and bioavailability of PTEs decrease in the following order: F1 > F2 > F3 > F4. In all cases, the most mobile fraction (F1) is reduced at least by half and immobilized as fractions of lower mobility, such as F3 and F4. 

Although the mobility of all studied PTEs decreased drastically, the mobility based on the % of PTEs in F1 followed the same trend as in the unamended soil Cd > Co > Ni > Zn = Cr > Cu > Pb. Moirou et al. [36] reported that in the case of Pb, Zn, and Cd, the major solubility decrease takes place between 0 and 10% zeolite addition, suggesting that the addition of higher amounts of zeolite content does not greatly enhance immobilization. However, Shanableh et al. [40] reported that optimum metal immobilization requests zeolite amendments up to 35% depending on the soil contamination level. 

In case of amendment with zeolites, the immobilization takes place by the increase of PTE fixation in the soil and consequent decrease of their mobility due to the increase of the soil pH, PTE diffusion to internal sorption sites in the zeolite structure, adsorption, surface precipitation, or redox processes [20]. In our case, no pH increase was observed after 30 days, and only a slight pH increase (<0.2 units) was noticed at 90 days after amendment. A possible explanation could be that the investigated soil sample already had a slightly alkaline pH (8.58) due to its calcite and dolomite content. The CEC increased with more than 10% at 30 days after amendment and reached an increase of about 25% at 90 the days after amendment, indicating that PTE diffusion into sorption sites and adsorption are the main immobilization mechanisms.

The immobilization effect of the 5% and 10% amendment is comparable, but a higher equilibration time enhances the immobilization effect, especially in the case of Cd, Co, Cu, Pb, and Zn. The increase of the equilibration time has no effect on the Cr and Ni mobility. Puyeo et al. [20] also reported the increase of Cd, Cu, and Zn immobilization over time in the case of mining-affected soils contaminated by an accidental spill from an opencast mine in Spain. The influence of the equilibration time on the immobilization effect could be due to the time necessary for the PTE migration and diffusion to internal sorption sites of the zeolites.

### 3.4. Environmental Risk Indicators

PTEs extracted in F1 are the most soluble and exchangeable, being readily available to biota, while those in F2 that are bound to hydrous oxides and amorphous ion/manganese oxides are less soluble than those in F1, but are mobilizable and may become available to biota. PTEs in F3 are bound to the organic matter and less soluble, and may be solubilized only under certain conditions, while those in F4 are associated with alumino-silicates and are considered to be inert and mobilizable only under extreme conditions [11,21,32]. Based on these considerations, several factors were developed to assess the environmental impact of PTE mobilization from soil, and consequently, the effect of metal immobilization by zeolite amendment.

#### 3.4.1. Individual and Global Contamination Factors

**Figure 3:**
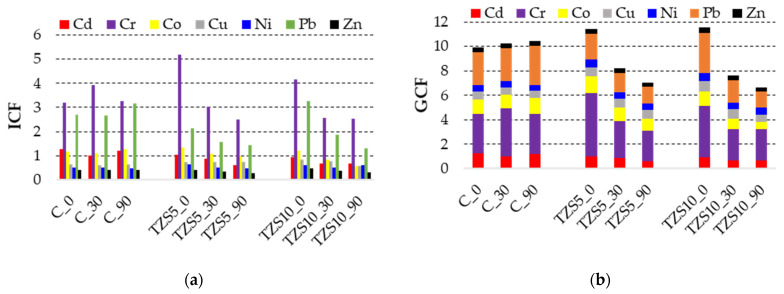
(**a**) Individual contamination factor (ICF) and (**b**) global contamination factor (GCF) of potentially toxic elements in the control soil (C) and soil amended with 5% (TZS5) and 10% (TZS10) zeolite at 0, 30, and 90 days after amendment.

The calculated ICF values (Figure 3a) indicated that the unamended soil has considerable contamination with Cr, moderate contamination with Cd, Pb, and Co, and low contamination of Cu, Ni, and Zn. After amendment, the Cr contamination becomes moderate; of Cd and Co, low; of Pb, remains moderate; and of Cu, Ni, and Zn, remains low.

The Cr and Pb have the highest ICF and thus the highest ability to be released, whereas the other PTEs have lower ICFs and are less prone to be mobilized. In the control soil, the ICFs are more or less constant during the equilibration time, indicating that the PTEs’ capacity to be released does not change. In the amended soils, the ICFs of Cr and Pb drastically decrease after 30 days and then remain more or less constant after 90 days of equilibration, while in the case of the other PTEs, the ICF decrease is less intense during the whole equilibration period. This fact suggests that Cr and Pb are more easily stabilized by the zeolite amendment than other PTEs, and that 30 days of equilibration time is enough to be stabilized. A possible explanation of the low stabilization efficiency of the other PTEs could be the transformation of the exchangeable fraction into oxidizable fraction in case of Co and Cu or the insufficient equilibration time.

The global contamination factor (GCF) indicated moderate contamination, and although after amendment the GCF value decreased, the global contamination level remained moderate in the case of both 5% and 10% amendments (Figure 3b).

#### 3.4.2. Risk Assessment Code

**Figure 4:**
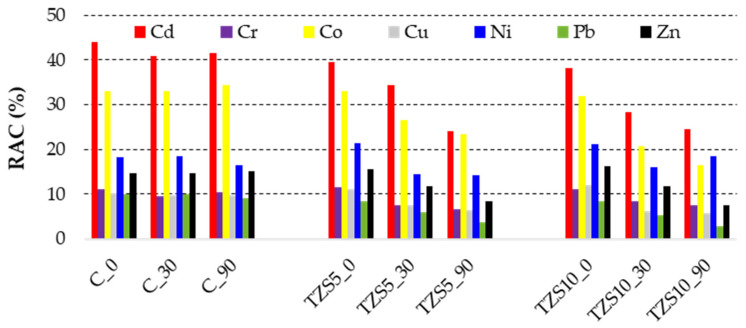
Risk assessment code (RAC) of potentially toxic elements in the control soil (C) and soil amended with 5% (TZS5) and 10% (TZS10) zeolite at 0, 30, and 90 days after amendment.

In the case of unamended soil, the RAC (Figure 4) indicated high risk for Cd and Co, medium risk for Ni, Zn, and Cr, and low risk for Cu and Pb.

In the case of Cd, after amendment with both 5 and 10% zeolite, the risk remained high after 30 days of equilibration and became medium after 90 days of equilibration, while in the case of Co, the risk decreased to medium after the first 30 days and remained medium even after 90 days of equilibration. The medium risk posed by Ni remained unchanged after amendments both after 30 and 90 days. The risk posed by Zn decreased from medium to low after 90 days of equilibration, while for that of Cr, after 30 days.

#### 3.4.3. Environmental Risk Factor (ERF)

**Figure 5:**
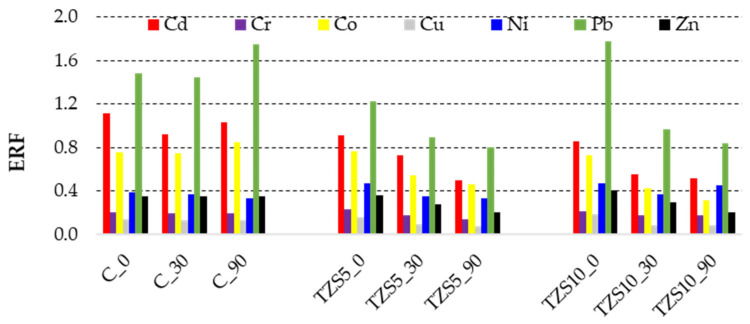
Environmental risk factor (ERF) of potentially toxic elements in the control soil (C) and soil amended with 5% (TZS5) and 10% (TZS10) zeolite at 0, 30, and 90 days after amendment.

The ERF values indicate low risk for Cr, Zn, and Cu, medium risk for Cd, Ni, Co, and high risk for Pb (Figure 5). After amendment, the risk of Co and Ni decreased from medium to low risk, while that of Cd did not change.

By amendment, the risk posed by Pb decreased from high to medium. This fact could be explained by the higher affinity of Pb to be immobilized by zeolites compared to the other elements.

## 4. Conclusions

In this study, the use of 5% and 10% thermally treated natural zeolite as soil amendment showed high potential for treating the industrially contaminated soils with potential toxic elements, due to the high content of clinoptilolite, high cation exchange capacity, and low PTE concentration. Generally, physico-chemical characteristics (pH, CEC, C_T_, N_T_, and humus) of zeolite-amended soils were not significantly influenced by the amendment ratio. The immobilization effect of the 5% and 10% amendment is similar; however, a higher equilibration time improves the immobilization effect, especially for Cd, Co, Cu, Pb, and Zn. Zeolite-amended soil equilibrated for 90 days showed significantly reduced PTE concentration in exchangeable and reducible fractions and increased PTE concentration in the oxidizable and residual fractions, particularly for Cd, Co, Cu, Pb, and Zn. Thus, the obtained results showed that the mobile and bioavailable fractions of investigated PTE were transformed into relatively stable fractions with the addition of zeolite. Additionally, the decrease of contamination factors, risk assessment code, and environmental risk factor support the immobilization effect of PTE by zeolite amendment. The soil amendments, such as thermally treated natural zeolites, can reduce the mobility, bioavailability, and toxicity of metals in soil. Therefore, the metal-contaminated sites can be amended with low quantities of natural zeolites, in order to ameliorate the overall ecosystems and to enhance plant growth and revegetation. Further experiments on natural zeolites should comprise long-term field application on various kinds of soil, and types and levels of metal contamination.

## Figures and Tables

**Table 1:** Physico-chemical properties of the raw (RZ) and thermally treated (TZ) zeolites and soil.

Parameter	RZ	TZ	Soil	Alert Threshold *	Intervention Threshold *
pH	9.52	9.55	8.58	-	-
Na_2_O (%)	1.61	1.69	0.14	-	-
K_2_O (%)	1.79	1.51	0.44	-	-
CaO (%)	3.61	3.64	19.1	-	-
MgO (%)	0.51	0.62	3.33	-	-
SiO_2_ (%)	69.96	69.68	-	-	-
Al_2_O_3_ (%)	13.61	14.02	2.40	-	-
Fe_2_O_3_ (%)	1.38	1.36	16.7	-	-
MnO (%)	0.03	0.04	0.50	-	-
TiO_2_ (%)	0.02	0.02	-	-	-
LOI (%)	7.47	7.38	-	-	-
Cd (mg/kg)	0.16	0.14	30.6	3	5
Cr (mg/kg)	4.29	4.14	17.7	100	300
Co (mg/kg)	3.19	3.03	23.2	30	50
Cu (mg/kg)	3.54	3.38	476	100	200
Ni (mg/kg)	5.44	4.91	19.0	75	150
Pb (mg/kg)	4.67	4.32	483	50	100
Zn (mg/kg)	15.9	15.5	3040	300	600
CEC (meq/100 g)	148	150	62.2	-	-
C_T_ (%)	<0.01	<0.01	2.82	-	-
N_T_ (%)	<0.01	<0.01	1.14	-	-
HA (%)	-	-	1.70	-	-

* Threshold for sensitive use according to Romanian legislation [35].

**Table 2:** Physico-chemical parameters of the control soil (C), 5% (TZS5), and 10% (TZS10) zeolite-amended soil at 0, 30, and 90 days.

Parameter	Time (Day)	C	TZS5	TZS10
pH (unit pH)	0	8.58	8.49	8.48
	30	8.60	8.66	8.64
	90	8.63	8.86	8.84
C (%)	0	2.82	2.70	2.56
	30	2.80	2.67	2.54
	90	2.85	2.65	2.52
N (%)	0	1.14	1.09	1.02
	30	1.10	1.08	1.01
	90	1.16	1.07	1.03
CEC (meq/100 g)	0	62.2	63.1	67.1
	30	62.0	69.7	71.8
	90	61.4	76.7	79.0
Humus (%)	0	1.70	1.69	1.33
	30	1.70	1.68	1.48
	90	1.74	1.63	1.54

**Table 3:** Potentially toxic element (PTE) concentrations in exchangeable and weak acid soluble fraction (F1), reducible fraction (F2), oxidizable fraction (F3), residual fraction (F4), and sum of F1–F4 (∑) in the control soil (C) and soil amended with 5% (TZS5) and 10% (TZS10) zeolite at 0, 30, and 90 days after amendment.

Amendment	Time (Day)	F	PTE (mg/kg)						
			Cd	Cr	Co	Cu	Ni	Pb	Zn
C	0	F1	12.8 ± 1.5	1.70 ± 0.18	7.26 ± 0.85	45.4 ± 5.5	3.06 ± 0.35	46.7 ± 5.5	413 ± 44
		F2	2.57 ± 0.34	0.92 ± 0.10	2.24 ± 0.29	7.44 ± 0.92	1.67 ± 0.23	233 ± 26	313 ± 35
		F3	0.90 ± 0.14	9.01 ± 1.12	2.42 ± 0.26	120 ± 15	1.02 ± 0.11	62.5 ± 7.0	83.9 ± 9.8
		F4	12.9 ± 1.50	3.62 ± 0.39	10.1 ± 1.2	272 ± 33	11.1 ± 1.4	126 ± 14	2007 ± 189
		**∑**	29.2 ± 3.8	15.2 ± 1.70	22.1 ± 2.6	445 ± 55	16.8 ± 2.0	469 ± 53	2817 ± 303
	30	F1	11.5 ± 1.0	1.50 ± 0.20	6.86 ± 0.55	40.8 ± 3.5	2.88 ± 0.29	42.2 ± 5.0	386 ± 37
		F2	2.00 ± 0.14	1.10 ± 0.22	2.00 ± 0.19	6.40 ± 0.76	1.35 ± 0.20	210 ± 25	302 ± 30
		F3	0.65 ± 0.12	10.0 ± 1.57	2.12 ± 0.29	108 ± 20	0.98 ± 0.10	58.9 ± 6.6	78.4 ± 8.8
		F4	14.0 ± 1.8	3.22 ± 0.52	9.80 ± 1.10	258 ± 30	10.4 ± 1.7	116 ± 16	1870 ± 140
		**∑**	28.2 ± 3.5	15.8 ± 2.6	20.8 ± 2.2	413 ± 54	15.6 ± 2.0	427 ± 52	2636 ± 253
	90	F1	12.4 ± 1.2	1.86 ± 0.22	7.56 ± 0.75	42.0 ± 4.5	2.76 ± 0.42	40.7 ± 4.8	408 ± 40
		F2	2.82 ± 0.44	1.05 ± 0.18	2.52 ± 0.33	7.14 ± 0.52	1.46 ± 0.13	243 ± 30	294 ± 27
		F3	1.1 ± 0.22	10.6 ± 0.9	2.33 ± 0.34	118 ± 20	1.14 ± 0.18	55.5 ± 7.2	81.5 ± 10.2
		F4	13.6 ± 1.1	4.12 ± 0.49	9.62 ± 1.22	266 ± 24	11.5 ± 1.5	107 ± 10	1910 ± 160
		**∑**	29.9 ± 4.2	17.6 ± 2.2	22.0 ± 2.8	433 ± 50	16.9 ± 2.3	446 ± 52	2693 ± 281
5%	0	F1	11.0 ± 1.0	1.68 ± 0.18	6.39 ± 0.67	42.4 ± 4.4	3.22 ± 0.34	37.4 ± 4.0	380 ± 41
		F2	2.30 ± 0.19	0.98 ± 0.19	2.00 ± 0.21	9.36 ± 1.05	1.58 ± 0.18	210 ± 24	267 ± 29
		F3	0.83 ± 0.09	9.41 ± 0.88	2.62 ± 0.27	108 ± 12	1.00 ± 0.2	58.1 ± 6.4	77.5 ± 8.1
		F4	13.7 ± 1.24	2.33 ± 0.25	8.29 ± 0.89	219 ± 23	9.20 ± 0.14	144 ± 16	1701 ± 184
		**∑**	27.8 ± 2.6	14.4 ± 1.9	19.3 ± 2.0	379 ± 41	15.0 ± 1.9	449 ± 50	2425 ± 260
	30	F1	8.91 ± 0.98	1.07 ± 0.12	5.03 ± 0.60	28.2 ± 2.9	2.02 ± 0.26	27.1 ± 3.0	290 ± 30
		F2	2.02 ± 0.24	1.10 ± 0.14	1.63 ± 0.14	3.64 ± 0.41	1.65 ± 0.19	184 ± 21	240 ± 25
		F3	1.28 ± 0.15	8.55 ± 1.08	3.21 ± 0.34	127 ± 14	1.18 ± 0.14	63.6 ± 7.2	91.0 ± 8.9
		F4	13.8 ± 1.5	3.54 ± 0.40	9.05 ± 0.94	217 ± 23	9.20 ± 1.1	173 ± 19	1838 ± 200
		**∑**	26.0 ± 2.4	14.3 ± 1.7	18.9 ± 2.0	377 ± 41	14.1 ± 1.7	448 ± 50	2458 ± 255
	90	F1	6.60 ± 0.72	0.99 ± 0.11	4.33 ± 0.39	22.0 ± 2.3	2.23 ± 0.25	15.9 ± 1.7	200 ± 18
		F2	2.54 ± 2.24	0.86 ± 0.10	1.49 ± 0.16	3.04 ± 0.32	1.63 ± 0.2	178 ± 20	207 ± 23
		F3	1.11 ± 0.10	8.78 ± 0.98	3.39 ± 0.35	120 ± 14	1.24 ± 0.23	63.0 ± 7.0	133 ± 16
		F4	17.2 ± 1.82	4.24 ± 0.45	9.35 ± 1.1	197 ± 22	10.5 ± 1.2	180 ± 21	1839 ± 208
		**∑**	27.4 ± 2.7	14.9 ± 1.7	18.6 ± 2.0	342 ± 38	15.6 ± 2.1	437 ± 49	2378 ± 260
10%	0	F1	10.3 ± 1.4	1.56 ± 0.19	5.92 ± 0.70	43.5 ± 5.4	2.99 ± 0.36	36.8 ± 4.8	393 ± 51
		F2	2.18 ± 0.32	0.88 ± 0.12	1.91 ± 0.26	13.1 ± 1.8	1.50 ± 0.18	242 ± 35	301 ± 38
		F3	0.72 ± 0.10	8.77 ± 1.10	2.39 ± 0.30	108 ± 15	0.91 ± 0.13	55.2 ± 7.1	73.7 ± 10.1
		F4	13.8 ± 1.8	2.70 ± 0.45	8.36 ± 1.21	196 ± 22	8.7 ± 1.2	102 ± 14	1635 ± 180
		**∑**	27.0 ± 3.7	13.9 ± 2.0	18.6 ± 2.4	361 ± 46	14.1 ± 1.8	436 ± 59	2402 ± 303
	30	F1	6.88 ± 0.92	1.21 ± 0.16	3.89 ± 0.51	23.4 ± 3.0	2.44 ± 0.31	24.0 ± 3.2	278 ± 35
		F2	1.76 ± 0.24	0.97 ± 0.12	1.64 ± 0.22	5.36 ± 0.83	1.64 ± 0.24	198 ± 25	254 ± 36
		F3	1.06 ± 0.2	8.18 ± 1.11	3.04 ± 0.41	137 ± 20	1.11 ± 0.15	72.5 ± 9.2	106 ± 12
		F4	14.6 ± 2.0	4.06 ± 0.54	10.1 ± 1.4	209 ± 26	10.0 ± 1.2	157 ± 24	1707 ± 310
		**∑**	24.3 ± 3.7	14.4 ± 1.9	18.7 ± 2.5	375 ± 52	15.2 ± 2.0	451 ± 61	2345 ± 335
	90	F1	6.25 ± 0.82	1.12 ± 0.11	3.26 ± 0.41	20.2 ± 2.6	2.34 ± 0.28	11.3 ± 1.2	179 ± 22
		F2	2.41 ± 0.30	1.08 ± 0.15	1.43 ± 0.18	6.88 ± 0.86	1.60 ± 0.21	177 ± 22	227 ± 31
		F3	1.69 ± 0.21	8.48 ± 1.03	2.60 ± 0.33	102 ± 14	0.90 ± 0.08	45.5 ± 7.2	162 ± 23
		F4	15.1 ± 2.0	4.19 ± 0.60	12.4 ± 1.7	226 ± 28	7.8 ± 1.1	179 ± 28	1841 ± 230
		**∑**	25.4 ± 3.3	14.9 ± 1.9	19.7 ± 2.5	355 ± 46	12.6 ± 1.5	413 ± 57	2409 ± 318
